# Positive detection of exfoliated colon cancer cells on linear stapler cartridges was associated with depth of tumor invasion and preoperative bowel preparation in colon cancer

**DOI:** 10.1186/s12957-016-0991-5

**Published:** 2016-08-31

**Authors:** Kishiko Ikehara, Shungo Endo, Kensuke Kumamoto, Eiji Hidaka, Fumio Ishida, Jun-ichi Tanaka, Shin-ei Kudo

**Affiliations:** 1 Digestive Disease Center, Showa University Northern Yokohama Hospital, 35-1 Chigasaki-chuo, Tsuzuki-ku, Yokohama, 224-8503 Japan; 2 Department of Coloproctology, Aizu Medical Center, Fukushima Medical University, 21-2 Tanisawa, Kawahigashi, Aizuwakamatsu, 969-3492 Japan; 3 Department of Surgery, Showa University Fujigaoka Hospital, 1-30 Fujigaoka, Aoba-ku, Yokohama, 227-8501 Japan

**Keywords:** Exfoliated cancer cell, Colon cancer, Functional end-to-end anastomosis, Suture-line recurrence

## Abstract

**Background:**

The aim of this study was to investigate exfoliated cancer cells (ECCs) on linear stapler cartridges used for anastomotic sites in colon cancer.

**Methods:**

We prospectively analyzed ECCs on linear stapler cartridges used for anastomosis in 100 colon cancer patients who underwent colectomy. Having completed the functional end-to-end anastomosis, the linear stapler cartridges were irrigated with saline, which was collected for cytological examination and cytological diagnoses were made by board-certified pathologists based on Papanicolaou staining.

**Results:**

The detection rate of ECCs on the linear stapler cartridges was 20 %. Positive detection of ECCs was significantly associated with depth of tumor invasion (*p* = 0.012) and preoperative bowel preparation (*p* = 0.003). There were no marked differences between ECC-positive and ECC-negative groups in terms of the operation methods, tumor location, histopathological classification, and surgical margins.

**Conclusions:**

Since ECCs were identified on the cartridge of the linear stapler used for anastomosis, preoperative mechanical bowel preparation using polyethylene glycol solution and cleansing at anastomotic sites using tumoricidal agents before anastomosis may be necessary to decrease ECCs in advanced colon cancer.

## Background

The cause of suture-line recurrence following curative colorectal cancer surgery is believed to be the presence of exfoliated cancer cells (ECCs) at the anastomotic site [1]. Many studies of suture-line recurrence have been conducted in rectal cancer surgery; however, a greater margin is used for colon cancer than for rectal cancer cases, so that suture-line recurrence is less common in colon cancer patients [2]. As a result, few studies have reported on the presence of ECCs in the colonic lumen of colon cancer patients. However, even in patients with colon cancer, the incidence of suture-line recurrence has been reported to be 0.8–5.9 % [2, 3]. Reconstruction following colon cancer surgery commonly involves the fabrication of a functional end-to-end anastomosis [4] using a linear stapler, as a convenient procedure [5]. In the present study, ECCs in the colonic lumen of colon cancer patients were examined based on the cytological examination of the solutions washing the linear stapler used for functional end-to-end anastomosis, and we further analyzed the relationship between positive detection of ECCs and clinicopathological factors and prognosis.

## Methods

### Patients

The study subjects were 100 consecutive patients who underwent laparoscopic (*n* = 61) or open (*n* = 39) colectomy for colon cancer at our hospital, in whom a functional end-to-end anastomosis using a linear stapler was performed. After completing the anastomosis in each case, the cartridge of the linear stapler was washed with saline, which was collected for cytological examination. Of the 100 patients examined, the localization of tumor was as follows: cecum 17 cases, ascending colon 41, transverse colon 28, descending colon 2, and sigmoid colon 12. The depth of tumor invasion was pTis 8 cases, pT1 16, pT2 7, pT3 47, and pT4 22. The histological diagnosis was well-differentiated adenocarcinoma 46 cases, moderately 42, poorly 7, and others 5. All patients were followed up 5 years or more after surgery, or until metastasis was developed or death. Written informed consent was obtained from all patients, after an explanation of the design and aim of this study.

### Preoperative bowel preparation

Full-laxative cleansing via polyethylene glycol solution (PEG) was routinely used; however, for patients with stenosis, other preparation methods (sodium picosulfate or enema) were employed.

### Cytology procedures

The procedure for the cytology of the washing samples collected from the stapler cartridges was as follows. After tumor resection, the intestinal lumen, distal and proximal to the anastomotic site, was cleaned five times using cotton balls soaked in either 5 % povidone-iodine or 0.025 % benzalkonium chloride. Afterwards, a functional end-to-end anastomosis was performed. The stapler cartridge (GIA 80-3.8, Covidien, MA, USA) used for anastomosis was washed using 100 ml of physiological saline, and the washing samples were promptly subjected to cytological analysis.

### Evaluation of data

Cytological diagnoses were made by board-certified pathologists based on Papanicolaou staining. Classes I, II, and III were included in the negative group, while classes IV and V were included in the positive group. Clinicopathological factors, including age, gender, operation methods (laparoscopic or open surgery), tumor location, depth of tumor invasion, tumor size, lymph node metastasis, distant metastasis, stage classification, histopathological classification, margin from tumor, and preoperative bowel preparation, and clinical outcome were compared between the two groups.

### Statistical analysis

In the present study, the *χ*^2^ test (Fisher’s exact test) was used to compare frequencies between the two groups, and the Mann-Whitney *U* test was used to compare intergroup differences. The level of significance was set at *p* < 0.05.

## Results

### Cytological analysis

The cytological analysis results were as follows: class I, *n* = 17; class II, *n* = 61; class III, *n* = 2; class IV, *n* = 7; and class V, *n* = 13 (Table 1). Based on the pathological diagnosis, all the patients were assigned to the positive group (*n* = 20) and the negative group (*n* = 80).

**Table 1:** Cytology from a cartridge of a stapler

Class I	17 cases (17 %)
Class II	61 (61 %)
Class III	2 (2 %)
Class IV	7 (7 %)
Class V	13 (13 %)

### The relationship between the status of ECCs and clinicopathological factors

All positive cases had advanced cancers that had invaded beyond the submucosal layer (T2–T4), while all of the early cancers that had invaded the submucosal layer (Tis, T1) were in the negative group (*p* = 0.012) (Table 2). Regarding preoperative bowel preparation, the detection rate of ECCs in cases where full-laxative mechanical cleansing with PEG was performed was significantly lower than that in cases where full-laxative mechanical cleansing was not possible (*p* = 0.003) (Table 2). The two groups did not differ significantly in terms of the operation method, tumor location, histopathological classification, and margin from tumor (Table 2).

**Table 2:** Comparison of clinicopathological factors in the ECC-positive and ECC-negative groups

	ECC-positive group	ECC-negative group	*p* value
	(*n* = 20)	(*n* = 80)	
Age	67.3 ± 12.0 years old	72.0 ± 9.6 years old	N.S
Gender			
M	8	43	N.S
F	12	37	
Operation method			
Laparoscopic surgery	10 (50 %)	51 (63.8 %)	N.S
Open surgery	10 (50 %)	29 (36.2 %)	
Tumor location			
Cecum	4 (20 %)	13 (16.3 %)	N.S
Ascending colon	9 (45 %)	32 (40 %)	
Transverse colon	4 (20 %)	24 (30 %)	
Descending colon	0 (0 %)	2 (2.5 %)	
Sigmoid colon	3 (15 %)	9 (11.2 %)	
Depth of tumor invasion			
pTis	0 (0 %)	8 (10 %)	0.012
pT1	0 (0 %)	16 (20 %)	(Tis, T1 vs. T2–T4)
pT2	2 (10 %)	5 (6.2 %)	
pT3	16 (80 %)	31 (38.8 %)	
pT4	2 (10 %)	20 (25 %)	
Tumor size (mean ± SE)			
	58.6 ± 16.7 mm	49.2 ± 32.6 mm	0.0239
Lymph node metastasis			
pN(−)	13	48	N.S
pN(+)	7	32	
Distant metastasis			
pM0	1	12	N.S
pM1	19	68	
cStage			
0	0	7	N.S
I	2	18	
II	10	21	
III	7	21	
IV	1	13	
Histopathological classification			
Well	8	38	N.S
Moderately	9	33	
Poorly	1	6	
Others	2	3	
Margin from tumor (mean ± SE)			
Oral side	16.7 ± 2.5 cm	14.6 ± 1.1 cm	N.S
Anal side	12.3 ± 1.5 cm	12.8 ± 1.0 cm	N.S
Preoperative bowel preparation			
PEG	8 (40 %)	60 (75 %)	0.003
Others	12 (60 %)	20 (25 %)	

### Follow-up outcome

Among all the cases, 81 were followed by colonoscopy after surgery. Follow-up colonoscopy was performed in 74 patients (74 %) (observation range 3.9–121.4 months; median observation period 64.1 months), and none were found to have developed suture-line recurrence.

## Discussion

We found that ECC-positive cases were recognized at 20 % on the stapler cartridges used for anastomosis in patients with colon cancer. According to the relationship between the status of ECCs and clinicopathological factors, the presence of ECCs was associated with depth of tumor invasion and preoperative bowel preparation. Our study included 86 patients with right-sided colon cancer in 100 colon cancer patients. Hasegawa et al. [6] reported that 2 (11.1 %) and 10 (55.6 %) of 18 patients, who underwent right hemicolectomy for right-sided colon cancer, had ECCs at the terminal ileum and distal colon anastomosis sites, respectively. They demonstrated that surgical bowel occlusion on both sides of the tumor before resection improved a decrease in the number of ECCs. Recently, Maeda et al. [7] have shown that ECCs were detected at distal colon and proximal colon of the tumor within each 3 cm in almost patients with sigmoid cancer, and ECCs decreased with distance from the tumor. They also suggested that bowel ligatures could decrease ECCs, leading to prevent local recurrence. These findings indicated that the no-touch isolation technique might influence the number of ECCs as previous reports suggested [8, 9]. In the present study, we performed colectomy according to the no-touch isolation technique. Consequently, the positive rate of ECCs was 20 %. The difference of positive rate might be explained by the operative technique in addition to other factors, including cleansing methods and bowel preparation methods.

Most studies of ECCs in the colonic lumen of colorectal cancer patients have involved rectal cancer patients. The incidence of suture-line recurrence has been reported to be 11–18 % [10, 11] in rectal cancer, while that in colon cancer has been reported to be 0.8–5.9 % [2, 3]. The possibility that ECCs can be implanted into freshly cut tissues was first postulated by Sir Ryall in 1907 [12]. This hypothesis was later advanced to explain some cases of suture-line recurrence following resection of colorectal cancer. In order to demonstrate the potential involvement of ECCs in the colonic lumen with suture-line and local recurrences in an animal model, Fermor et al. [13] injected ECCs collected from the colonic lumen of 17 colorectal cancer patients into the caudal vein of immunocompromised mice and showed that six mice developed lung metastases. Several cases of suture-line recurrence following resection of colon cancer, especially sigmoid colon, have been reported in Japan [14–17]. They discussed that implantation was considered to be the most important factor in terms of the cause of recurrence at anastomotic sites. The frequency of suture-line recurrence in colon cancer was very low though ECCs were frequently detected in colonic lumen. Indeed, we detected ECCs in 20 % of the colon cancer cases we analyzed. However, no suture-line recurrence was observed in our series of colon cancer for 5-year observation after colectomy. The cell viability of ECCs was investigated to understand etiological mechanism of anastomotic recurrence. Rosenberg et al. [18] reported the presence of no viable cells, as assessed by trypan blue exclusion. However, Umpleby et al. [19], using trypan blue exclusion and hydrolysis of fluorescein diacetate, showed that 70 % of patients with colorectal cancer had viable cells exfoliated into the bowel lumen. In general, other factors, which are associated with the potential of implantation, must be required even if ECCs still have viability.

In the present study, we found that the positive rate of ECCs was 11.8 % in patients with PEG, while it was 37.5 % in patients without PEG. Mechanical bowel preparation using PEG may be effective for decreasing the number of ECCs. Previous reports [6, 7] also suggested same findings. Regarding mechanical bowel preparation using PEG, Slim et al. [20] have described that anastomotic leakage was significantly found after mechanical bowel preparation (odds ratio 1.75; *p* = 0.032) and concluded that mechanical bowel preparation using PEG should be omitted before elective colorectal surgery. Therefore, mechanical bowel preparation is routinely not performed in patients undergoing elective colon cancer because of employing “enhanced recovery after surgery” (ERAS) protocol widely. Recently, clinical practice guidelines [21] from the USA recommend the use of both mechanical bowel preparation and oral antibiotic bowel preparation. Many surgeons still conduct mechanical bowel preparation using PEG in the USA [22] and Japan [23]. Mechanical bowel preparation using PEG might contribute to decrease positive ECCs in advanced colorectal cancer, leading to decrease the possibility of anastomotic recurrence in addition to prevention of anastomotic leakage and surgical site infection.

In a previous study [5], the outcome of reconstruction following colon cancer surgery was compared between hand-sewn and stapled anastomosis. While the safety was comparable, it was radiographically confirmed that suture leakage was more common with hand-sewn anastomoses. Hence, functional end-to-end anastomosis has primarily been performed using a stapler. In the present study, the washing samples of the cartridge of the linear stapler used for functional end-to-end anastomosis were examined cytologically. The rationale for using the washing samples of the cartridge in this study was that they would better reflect the presence of ECCs than the washing solutions of the colonic lumen. In addition, the presence of ECCs on the cartridge was considered to be a cause of suture-line recurrence. Prior to the present study, ECCs had been cytologically identified in the physiological saline used to irrigate the colonic lumen. In the present study, 5 % povidone-iodine was first used as a tumoricidal agent to irrigate the colonic lumen prior to anastomosis [24, 25]. However, since povidone-iodine should not be used in the abdominal cavity due to severe damage of normal mucosa, we have employed 0.025 % benzalkonium chloride, as an alternative. Therefore, the ECC detection rate might be lower in the present study than that reported by Umpleby, et al. [19] who used normal saline for bowel irrigation.

Our results indicated that the detection rate of ECCs, which could be detected at anastomotic sites even on the stapler cartridges, might be increased in advanced colon cancer with more than T2 and in elective colon cancer patients without mechanical bowel preparation using PEG, leading to increase the possibility of incidence of suture-line recurrence. To prevent the possibility of incidence of suture-line recurrence, we should conduct preoperative mechanical bowel preparation using PEG, perform surgical operation by the no-touch isolation technique, and irrigate the colonic lumen prior to anastomosis using 0.025 % benzalkonium chloride as a tumoricidal agent.

## Conclusions

We found ECCs on the cartridge of the linear stapler used for anastomosis in 20 % of the colon cancer cases we analyzed. Most of the positive ECCs were identified in advanced colon cancer without PEG. Therefore, preoperative mechanical bowel preparation using PEG and cleansing at anastomotic sites using tumoricidal agents before anastomosis may contribute to decrease ECCs in advanced colon cancer.

## Abbreviations

ECCs: Exfoliated cancer cells
PEG: Polyethylene glycol solution

## References

[1] Gologher JC, Dukes CE, Bussey HJR (1951). Local recurrences after sphincter saving excisions for carcinoma of the rectum and rectosigmoid. Br J Surg.

[2] Hardy KJ, Cuthbertson AM, Hughes ES (1971). Suture-line neoplastic recurrence following large-bowel resection. Aust NZ J Surg.

[3] Kyzer S, Gordon PH (1992). The stapled functional end-to-end anastomosis following colonic resection. Int J Colorectal Dis.

[4] Steichen FM (1968). The use of staplers in anatomical side-to-side and functional end-to-end enteroanastomoses. Surgery.

[5] Docherty JG, McGregor JR, Akyol AM, Murray GD, Galloway DJ (1995). Comparison of manually constructed and stapled anastomoses in colorectal surgery. West of Scotland and Highland anastomosis Study Group. Ann Surg.

[6] Hasegawa J, Nishimura J, Yamamoto S, Yoshida Y, Iwase K, Kawano K (2011). Exfoliated malignant cells at the anastomosis site in colon cancer surgery: the impact of surgical bowel occlusion and intraluminal cleaning. Int J Colorectal Dis.

[7] Maeda K, Hanai T, Sato H, Masumori K, Koide Y, Matsuoka H (2014). Intraluminal exfoliated cancer cells and effectiveness of bowel ligatures during sigmoidectomy for sigmoid colon cancer. Surg Today.

[8] Slanetz CA (1998). Effect of no touch isolation on survival and recurrence in curative resections for colorectal cancer. Ann Surg Oncol.

[9] Hayashi N, Egami H, Kai M, Kurusu Y, Takano S, Ogawa M (1999). No-touch isolation technique reduces intraoperative shedding of tumor cells into the portal vein during resection of colorectal cancer. Surgery.

[10] Phillips RK, Hittinger R, Blesovsky L, Fry JS, Fielding LP (1984). Local recurrence following ‘curative’ surgery for large bowel cancer: II. The rectum and rectosigmoid. Br J Surg.

[11] Hojo K (1986). Anastomotic recurrence after sphincter-saving resection for rectal cancer. Length of distal clearance of the bowel. Dis Colon Rectum.

[12] Ryall C (1907). Cancer infection and cancer recurrence. Lancet.

[13] Femor B, Umpleby HC, Lever JV, Symes MO, Williamson RCN (1986). Proliferative and metastatic potential of exfoliated colorectal cancer cells. J Natl Cancer Inst.

[14] Yamauchi T, Shida D, Tanizawa T, Inada K (2013). Anastomotic recurrence of sigmoid colon cancer over five years after surgery. Case Rep Gastroenterol.

[15] Miyake H, Moriya Y, Maruyama K, Yokota T, Shimoda T (1998). Anastomotic recurrence after curative resection of a transverse colon carcinoma: a case report. Jpn J Clin Oncol.

[16] Matsumoto M, Maruta M, Maeda K, Utsumi T, Tohyama K, Msumori K (1999). Suture line recurrence following curative resection for carcinoma of the colon-report of two cases. J Jap Surg Assoc.

[17] Tsunoda A, Kawamura M, Nakao K, Yoshizawa H, Kawaguchi T, Marumori K (1993). Recurrence at the suture line following resection for carcinoma of the colon. J Jap Coloproctol.

[18] Rosenberg IL, Russell CW, Giles GR (1978). Cell viability studies on the exfoliated colonic cancer cell. Br J Surg.

[19] Umpleby HC, Fermor B, Symes MO, Willamson RCN (1984). Viability of exfoliated colorectal carcinoma cells. Br J Surg.

[20] Slim K, Vicaut E, Panis Y, Chipponi J (2004). Meta-analysis of randomized clinical trials of colorectal surgery with or without mechanical bowel preparation. Br J Surg.

[21] Bratzler DW, Dellinger EP, Olsen KM, Perl TM, Auwaerter PG, Bolon MK (2013). Clinical practice guidelines for antimicrobial prophylaxis in surgery. Surg Infect (Larchmt).

[22] Zmora O, Wexner SD, Hajjar L, Park T, Efron JE, Nogueras JJ (2003). Trends in preparation for colorectal surgery: survey of the members of the American Society of Colon and Rectal Surgeons. Am Surg.

[23] Kobayashi M, Takesue Y, Kitagawa Y, Kusunoki M, Sumiyama Y (2011). Antimicrobial prophylaxis and colon preparation for colorectal surgery: results of a questionnaire survey of 721 certified institutions in Japan. Surg Today.

[24] Basya G, Penninckx F, Mebis J, Filez L, Geboes K, Yap P (1999). Local and systemic effects of intraoperative whole-colon washout with 5 per cent povidone-iodine. Br J Surg.

[25] Tsunoda A, Shibusawa M, Tsunoda Y, Choh H, Takata M, Kusano M (1997). Implantation on the suture material and efficacy of povidone-iodine solution. Eur Sur Res.

